# Does Cannabis Legalization Contribute to an Increase in the Incidence and Prevalence of Psychotic Disorders?

**DOI:** 10.1192/j.eurpsy.2025.255

**Published:** 2025-08-26

**Authors:** D. Quattrone

**Affiliations:** Social, Genetic, and Developmental Psychiatry Centre - Institute of Psychiatry, Psychoolgy and Neuroscience, King’s College London, London, United Kingdom

## Abstract

**Abstract:**

Cannabis legalisation has undergone a rapid global transformation, with varying policy approaches and public health implications across different geographic areas and social contexts.

Several arguments have been proposed to support cannabis legalisation, ranging from control of the quality and potency of the market products, harm reduction, addressing the black market activities, reducing crime, and economic benefit. However, cannabis use has been associated with public health concerns, and it has been established as the most preventable risk factor for psychotic disorders.

This work aims to dissect the key arguments supporting cannabis legalisation through the following objectives: 1) reviewing the relationship between cannabis legalisation and the incidence and prevalence of psychotic disorders in countries where cannabis has been legalised, as well as changes in incidence rates over time in those countries where legalization is currently under debate; 2) examining the relationship between cannabis use and psychopathological outcomes using a syndemic approach; and 3) presenting original data from the epidemiological branch of the EC-1 study, aiming to identify risk factors for psychopathology, violence, and aggression in South London.

**Disclosure of Interest:**

None Declared

